# Impact of reconstruction parameters on the accuracy of myocardial extracellular volume quantification on a first-generation, photon-counting detector CT

**DOI:** 10.1186/s41747-024-00469-7

**Published:** 2024-06-19

**Authors:** Chiara Gnasso, Daniel Pinos, U. Joseph Schoepf, Milan Vecsey-Nagy, Gilberto J. Aquino, Nicola Fink, Emese Zsarnoczay, Robert J. Holtackers, Jonathan Stock, Pal Suranyi, Akos Varga-Szemes, Tilman Emrich

**Affiliations:** 1 Division of Cardiovascular Imaging, Department of Radiology and Radiological Science, Medical University of South Carolina, 25 Courtenay Dr, Charleston, SC 29425 USA; 2 Experimental Imaging Center, IRCCS San Raffaele Scientific Institute, Via Olgettina 60, Milan, Italy; 3 Heart and Vascular Center, Semmelweis University, Varosmajor Utca 68, Budapest, 1122 Hungary; 4 Department of Radiology, University Hospital, LMU Munich, Marchioninistr. 15, Munich, 81377 Germany; 5 MTA-SE Cardiovascular Imaging Research Group, Department of Radiology, Medical Imaging Centre, Semmelweis University, Üllői Út 78, Budapest, 1082 Hungary; 6 Department of Radiology and Nuclear Medicine, Maastricht University Medical Centre, Maastricht, 6229 HX The Netherlands; 7 Cardiovascular Research Institute Maastricht (CARIM), Maa stricht University, Maastricht, 6229 ER The Netherlands; 8 Paracelsus Medical University, Prof.-Ernst-Nathan-Strasse 1, Nuremberg, 90419 Germany; 9 Department of Diagnostic and Interventional Radiology, University Medical Center of Johannes Gutenberg-University, Langenbeckstr. 1, Mainz, 55131 Germany; 10 German Centre for Cardiovascular Research, Mainz, 55131 Germany

**Keywords:** Fibrosis, Magnetic resonance imaging, Myocardium, Tomography (x-ray computed)

## Abstract

**Background:**

The potential role of cardiac computed tomography (CT) has increasingly been demonstrated for the assessment of diffuse myocardial fibrosis through the quantification of extracellular volume (ECV). Photon-counting detector (PCD)-CT technology may deliver more accurate ECV quantification compared to energy-integrating detector CT. We evaluated the impact of reconstruction settings on the accuracy of ECV quantification using PCD-CT, with magnetic resonance imaging (MRI)-based ECV as reference.

**Methods:**

In this post hoc analysis, 27 patients (aged 53.1 ± 17.2 years (mean ± standard deviation); 14 women) underwent same-day cardiac PCD-CT and MRI. Late iodine CT scans were reconstructed with different quantum iterative reconstruction levels (QIR 1−4), slice thicknesses (0.4−8 mm), and virtual monoenergetic imaging levels (VMI, 40−90 keV); ECV was quantified for each reconstruction setting. Repeated measures ANOVA and *t*-test for pairwise comparisons, Bland–Altman plots, and Lin’s concordance correlation coefficient (CCC) were used.

**Results:**

ECV values did not differ significantly among QIR levels (*p* = 1.000). A significant difference was observed throughout different slice thicknesses, with 0.4 mm yielding the highest agreement with MRI-based ECV (CCC = 0.944); 45-keV VMI reconstructions showed the lowest mean bias (0.6, 95% confidence interval 0.1–1.4) compared to MRI. Using the most optimal reconstruction settings (QIR4. slice thickness 0.4 mm, VMI 45 keV), a 63% reduction in mean bias and a 6% increase in concordance with MRI-based ECV were achieved compared to standard settings (QIR3, slice thickness 1.5 mm; VMI 65 keV).

**Conclusions:**

The selection of appropriate reconstruction parameters improved the agreement between PCD-CT and MRI-based ECV.

**Relevance statement:**

Tailoring PCD-CT reconstruction parameters optimizes ECV quantification compared to MRI, potentially improving its clinical utility.

**Key points:**

• CT is increasingly promising for myocardial tissue characterization, assessing focal and diffuse fibrosis via late iodine enhancement and ECV quantification, respectively.

• PCD-CT offers superior performance over conventional CT, potentially improving ECV quantification and its agreement with MRI-based ECV.

• Tailoring PCD-CT reconstruction parameters optimizes ECV quantification compared to MRI, potentially improving its clinical utility.

**Graphical Abstract:**

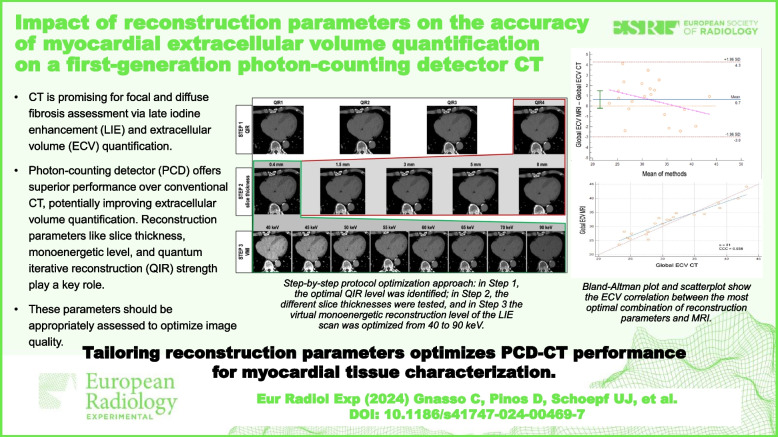

## Background

The detection of myocardial scar and fibrosis has diagnostic and prognostic relevance, especially since the introduction of antifibrotic therapies [1, 2]. Magnetic resonance imaging (MRI) is the current noninvasive reference standard for myocardial viability assessment [3], typically based on late gadolinium enhancement, allowing the assessment of focal fibrosis and scars, as well as T1 mapping sequences, which allow the assessment of diffuse, reactive, and interstitial fibrosis through the quantification of extracellular volume (ECV) [4]. Over the last decade, CT has increasingly demonstrated its potential for myocardial tissue characterization, primarily for the evaluation of focal fibrosis through late iodine enhancement (LIE) and later for the assessment of diffuse fibrosis using ECV quantification [5, 6]. CT-based scar detection and ECV quantification are not yet routinely applied, even though their value has already been demonstrated in various clinical scenarios [7–9].

The strength of CT-based ECV quantification relies on its high inter- and intra-observer reproducibility and the ability to visualize coronary anatomy during the same session [10]. Dual-energy CT can further improve ECV assessment compared to single‐energy by enabling the creation of iodine maps. Iodine maps automatically and accurately quantify ECV, eliminating the need for true noncontrast acquisitions, thus reducing radiation exposure [11]. Conventional energy-integrating detectors (EID)-CT, however, demonstrate a slight systematic overestimation of ECV compared to MRI [12].

The recently introduced photon-counting detector (PCD)-CT technique offers several advantages over EID-CT such as the availability of spectral data without temporal resolution penalty and improved spatial and contrast resolution [13]. These advancements have the potential to improve ECV-based myocardial tissue characterization, which has been demonstrated to correlate strongly with MRI-ECV when using EID-CT [14, 15]. We hypothesized that an optimal combination of reconstruction parameters improves ECV quantification accuracy and can reduce such overestimation.

Therefore, the aim of this study was to evaluate the impact of CT image reconstruction settings on the accuracy of ECV quantification using a first-generation PCD-CT, with MRI-ECV as reference.

## Methods

### Study population

This post hoc analysis was performed based on a previously published prospective study [15]. The protocol of the prospective, observational, single-center, Health Insurance Portability and Accountability Act–compliant study was approved by the local institutional review board (Number: *Pro00108359*). Written informed consent was obtained for all participants. From July 2021 to January 2022, consecutive patients undergoing a clinically indicated cardiac MRI were enrolled and underwent a research cardiac CT on the same day. Inclusion criteria were (i) age ≥ 18 years and (ii) clinical indication for cardiac MRI. Exclusion criteria were (i) refusal to consent and (ii) contraindication to iodine-based contrast media (positive anamnesis of allergy to iodinated contrast media or impaired renal function, defined as a creatinine level > 1.5 mg/dL). Demographic information, medical history, and laboratory values were extracted from electronic medical records.

### MRI acquisition protocol

Cardiac MRI was performed on a 1.5-T system (MAGNETOM Avanto; Siemens Healthineers, Erlangen, Germany) using a dedicated electrocardiogram (ECG)-gated acquisition protocol including myocardial native and postcontrast T1 mapping. T1 mapping images of the left ventricle (LV) were acquired in a two-chamber short-axis view at basal, midventricular, and apical positions using a modified Look-Locker (MOLLI) inversion-recovery sequence with a 5(3)3 sampling scheme with the following pulse sequence parameters: repetition time/echo time 2.6/1.1 ms; field of view 300 × 256 mm^2^; slice thickness 8 mm; image acquisition matrix 192 × 128; reconstruction matrix 192 × 164; in-plane spatial resolution 1.56 × 1.56 mm^2^; bandwidth 1,085 Hz/pixel; flip angle 35°; and parallel imaging acceleration factor. T1 mapping acquisition was repeated at the equilibrium phase at 10–12 min after the administration of 0.1 mmol/kg gadobutrol (Gadavist; Bayer Healthcare, Berlin, Germany), at the same slice positions as for native T1 mapping, using a MOLLI 4(1)3(1)2 sampling scheme.

### MRI postprocessing and ECV analysis

All cardiac MRI examinations were analyzed using dedicated cardiac software (Circle cvi42 v.5.12.2, Circle Cardiovascular Imaging, Calgary, Canada). The LV myocardial wall and the intra-ventricular blood pool were segmented on native and post-contrast T1 maps. The two maps were then coregistered using a contour-based registration method, and ECV was calculated as follows:

$$\mathrm{ECV }= (1 -\mathrm{ Hct}) \times \frac{\left(\frac{1}{{{\text{T}}1}_{\mathrm{myo\ post}}} - \frac{1}{{{\text{T}}1}_{\mathrm{myo\ pre}}}\right)}{\left(\frac{1}{{{\text{T}}1}_{\mathrm{blood\ post }}} - \frac{1}{{{\text{T}}1}_{\mathrm{blood\ pre}}}\right)}$$

where Hct is the hematocrit, T1_myo pre_ and T1_myo post_ are native and postcontrast T1 measurements in the myocardium, respectively, while T1_blood pre_ and T1_blood post_ are native and post-contrast T1 measurements in the LV blood compartment, respectively.

Partial volume averaging due to intraventricular blood and epicardial fat was mitigated by setting an automatic offset of 25% from the LV endocardial and epicardial borders. Since laboratory-based Hct measurements were not available for all patients, a synthetic Hct value was calculated from the native T_1_ maps following a locally established formula [16]:

$$\mathrm{Synthetic\, Hct}= \left(898\times \left[\frac{1}{{{\text{T}}1}_{{\text{blood}}}}\right]\right)- 0.16$$

where T1_blood_ is the native T1 measured at the LV.

### CT acquisition protocol

All CT scans were performed on a first-generation dual-source PCD-CT system (NAEOTOM Alpha; Siemens Healthineers, Forchheim, Germany) equipped with two cadmium telluride PCDs, each with a 144 × 0.4-mm collimation. The gantry rotation time was 0.25 s. Tube voltage was set at 120 kVp per vendor recommendations, while the tube current was automatically adjusted to reach the chosen image quality level (CARE Dose4D, Siemens Healthineers). Coronary CT angiography (CCTA) was performed after the intravenous administration of 100 mL iopromide (Ultravist, 370 mg I/mL; Bayer Healthcare, Berlin, Germany) at a flow rate of 5–6 mL/s along with a 20-mL saline chaser. The image quality level for automated tube voltage selection (CARE kV, Siemens Healthineers) for the CCTA was set to 44 (equivalent to a standard CCTA scan), and an ECG-triggered window was set from 30 to 80% of the RR interval. Scan parameters were aligned to a recently published protocol [14]. LIE scans were acquired using a prospective ECG-triggered protocol with a fixed 280-ms delay from the R-wave, 5 min after contrast administration. CARE kV was set to an image quality level of 55, equivalent to a standard LIE cardiac scan. All scans were performed using a standard Quantum Plus (Siemens Healthineers) acquisition mode, which allows spectral imaging-based iodine map reconstructions.

### CT image reconstruction and ECV analysis

CCTA and LIE scans were reconstructed with the use of a proprietary offline image reconstruction platform (ReconCT, version 15.0.58757.0, Siemens Healthineers). The LIE scans were used to generate iodine maps. ECV analysis was performed using a prototype software (CT Cardiac Functional Analysis, version 2.0.9, Siemens Healthineers), as follows: in a preprocessing step, CCTA, LIE-based virtual monoenergetic images (VMI) and iodine maps were aligned by nonrigid registration with the reference coordinate frame of the CCTA to enable accurate segmentation of the myocardium and blood pool. For the ECV quantification, iodine maps associated with the LIE scan contained all the information needed, and ECV was calculated as follows:

$$\mathrm{ECV }= (1-{\text{Hct}}) \times \frac{\mathrm{Iodine\, density\, myocardium}}{\mathrm{Iodine\, density\, bloodpool}}$$

The same synthetic Hct value was used as for the MRI-based ECV quantification. For quantitative analysis, the mid-myocardial wall was considered, using the same 25% offset as in MRI. ECV was calculated for the entire LV volume, and the American Heart Association polar maps with 17 myocardial segments were automatically computed and displayed [17].

**Fig. 1:**
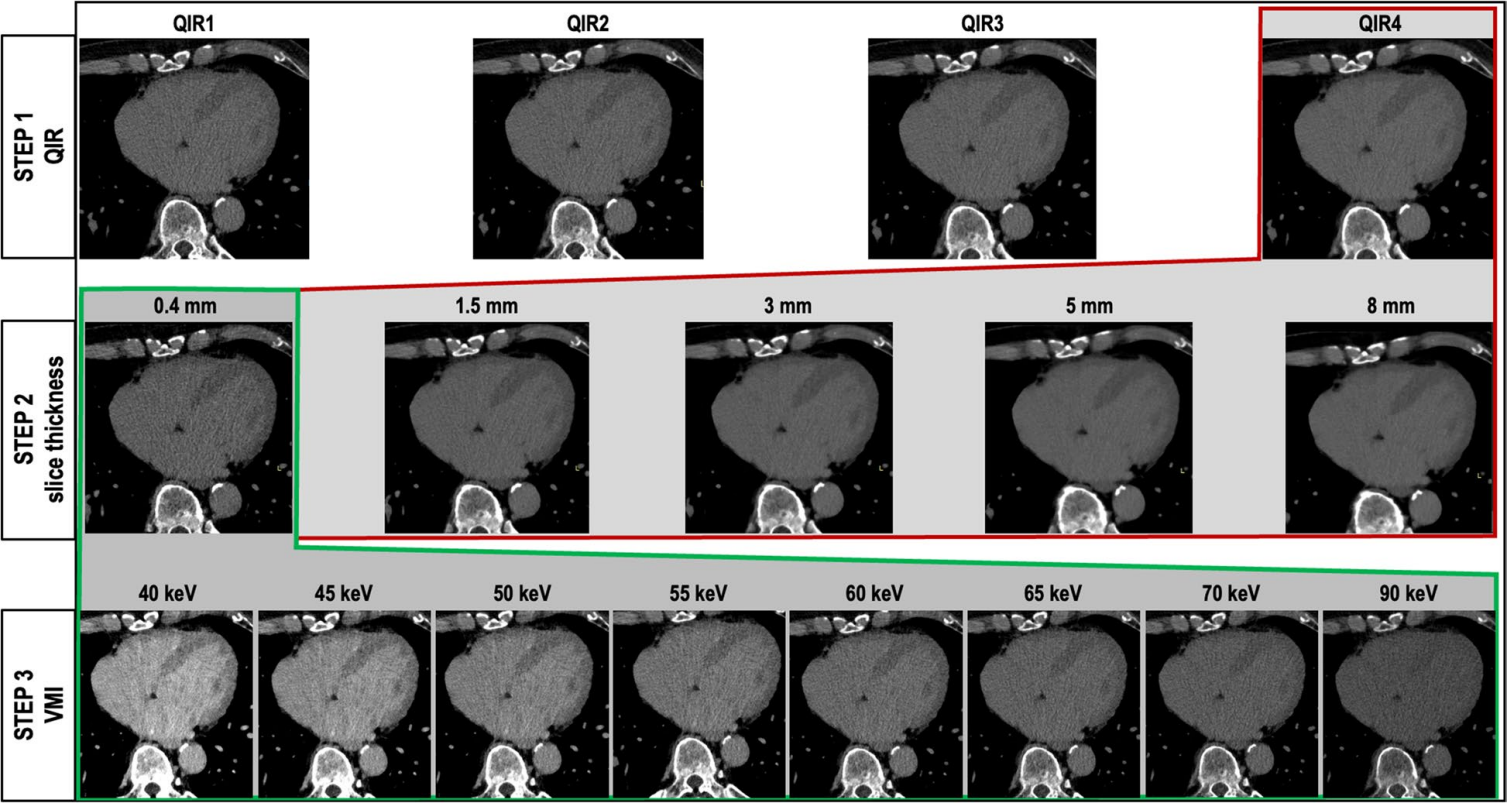
Representation of the systematic step-by-step protocol optimization approach. In Step 1, the optimal quantum iterative reconstruction level (14) was identified. In Step 2, the different slice thicknesses were tested from 0.4 to 8 mm. In Step 3, the virtual monoenergetic image reconstruction level of the late iodine enhancement scan was optimized from 40 to 90 keV. The parameter with the best diagnostic performance in each step was retained in the subsequent step

To find the optimal combination of reconstruction parameters with the best concordance with MRI, a step-by-step iterative process was chosen in which only one CT reconstruction parameter was modified at a time. ECV was calculated each time one of the reconstruction parameters was changed. The baseline reconstruction setting was the same used in current literature for ECV quantification on a PCD-CT scanner [14, 15]: slice thickness 1.5 mm; increment 1 mm; quantum iterative reconstruction (QIR) 3; kernel Qr40; and LIE VMI reconstructed at 65 keV. In Step 1, the QIR level was modified, obtaining four sets of images ranging from QIR 1 to QIR 4. In Step 2, different slice thicknesses/increments were tested, namely: 0.4/0.3, 1.5/1.0, 3.0/2.5, 5.0/4.0, and 8.0/7.0 mm. In Step 3, VMI levels of the LIE scan were adjusted, including 40, 45, 50, 55, 60, 65, 70, and 90 keV. The parameter with the best tradeoff between the highest concordance and lowest bias compared to MRI was retained in each step for the subsequent step. A visual representation of this step-by-step approach is illustrated in Fig. 1.

### Statistical analysis

Statistical analyses were performed with MedCalc, version 20.110 (MedCalc Software Ltd, Ostend, Belgium). Normality of data was assessed using the Shapiro–Wilk test. Categorical variables were reported as absolute values and percentages, while continuous variables as mean ± standard deviation. At each step, the CT-based ECV quantifications derived from different combinations of reconstruction parameters were compared using the ANOVA test for repeated measures and the *t*-test for pairwise comparisons. Lin’s concordance correlation coefficient (CCC) was calculated to measure the agreement among the different CT-based ECV values and the MRI-based ECV, which was used as reference standard. CCC values were interpreted as follows: correlation < 0.200 = poor agreement; 0.200−0.399 = fair agreement; 0.400−0.599 = moderate agreement; 0.600−0.799 = good agreement; and 0.800−1.000 = excellent agreement [18]. Bland–Altman plots were used to visualize the agreement between CT- and MRI-based ECV quantification with ± 1.96 standard deviations limits of agreement (LoA) and to identify bias between the two measurements. No data imputation for missing values was used. All tests were two-tailed, and a *p*-value < 0.05 was considered statistically significant. Bonferroni correction was applied when multiple testing was performed.

## Results

### Population characteristics

Twenty-seven patients were enrolled in this study and underwent cardiac MRI and CT on the same day. Demographic and clinical characteristics, as well as ECV quantification with MRI and baseline CT, are detailed in Table 1. The mean global MRI-based ECV was 31.1 ± 5.9% and was available only in 21/27 (77.8%) patients since not all subjects had all three myocardial slices available with adequate image quality. The baseline mean global CT-based ECV was 33.2 ± 4.7% and was available in all patients.

**Table 1:** Baseline patient characteristics

Patient characteristics	Value
Male sex (*n*, %)	13/27 (48.1%)
Age (years)	53.1 ± 17.2
Body mass index (kg/m^2^)	28.6 ± 5.3
Smoking history (*n*, %)	1/27 (3.7%)
Hypertension (*n*, %)	17/27 (63%)
Nonischemic cardiomyopathy (*n*, %)	12/27 (44.4%)
Congestive heart failure (*n*, %)	10/27 (37%)
Aortic/mitral valve disease (*n*, %)	7/27 (25.9%)
Coronary artery disease (*n*, %)	6/27 (22.2%)
Diabetes (*n*, %)	3/27 (11.1%)
Dyslipidemia (*n*, %)	9/27 (33.3%)
MRI-based ECV basal (*n* = 22) (%)	30.6 ± 5.6
MRI-based ECV midventricular (*n* = 21) (%)	30.3 ± 5.7
MRI-based ECV apical (*n* = 21) (%)	33 ± 7.3
MRI-based ECV global (*n* = 21) (%)	31.1 ± 5.9
CT-based ECV basal (*n* = 27) (%)	32.9 ± 5.3
CT-based ECV midventricular (*n* = 27) (%)	32.6 ± 4.6
CT-based ECV apical (*n* = 27) (%)	34.3 ± 5.6
CT-based ECV global (*n* = 27) (%)	33.2 ± 4.7

Values are means ± standard deviation or frequencies with percentages. Baseline reconstruction parameters were quantum iterative reconstruction level 3, slice thickness 1.5 mm, and virtual monoenergetic image at 65 keV

*CT* Computed tomography, *ECV* Extracellular volume, *MRI* Magnetic resonance imaging

### Step 1: QIR optimization

Statistical analysis did not show any significant difference among CT-based ECV values when changing the QIR strength level. Although ECV values were comparable, a slight tendency for better quantification accuracy was observed when increasing the QIR level, as demonstrated by the CCC and the mean bias in the Bland–Altman plot. The concordance between CT- and MRI-based ECV was 0.87 for images reconstructed with a QIR 1, and 0.89 for images reconstructed with a QIR 4; the mean bias was 1.7 (95% confidence interval (CI) 0.8–2.6, LoA 2.3–5.8) with QIR 1, and 1.6 (95% CI 0.7–2.5, LoA -2.2–5.5) using QIR 4. In all the cases, CT demonstrated a systematic overestimation of ECV with respect to MRI.

### Step 2: slice thickness optimization

**Fig. 2:**
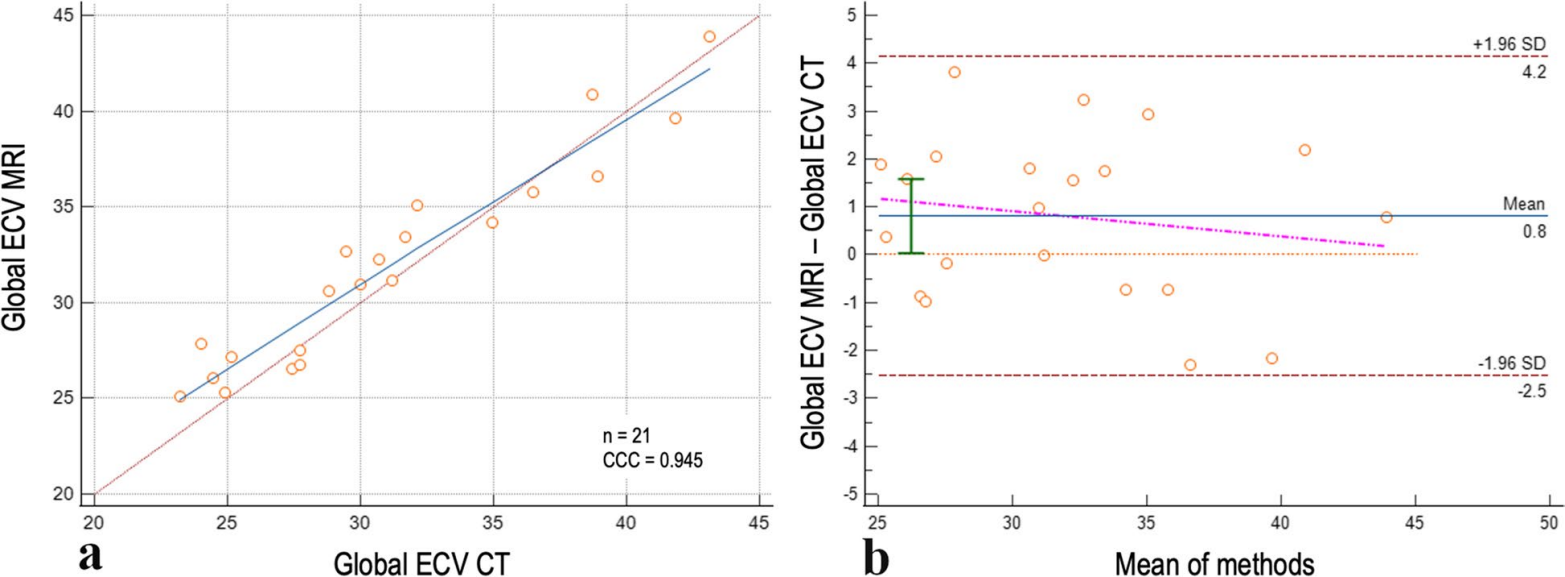
Correlation of ECV between CT and MRI using 0.4-mm slice thickness. Scatterplot (**a**) and Bland–Altman plot (**b**) showing the global ECV correlation between CT and MRI when using QIR 4, slice thickness/increment 0.4/0.3 mm, and VMI 65 keV. Horizontal lines are drawn at the mean difference (blue), and the limits of agreement (red); in the graph are also displayed the line of equality (orange horizontal line), the regression line of differences (pink line), and the 95% confidence interval of mean difference (green bar). *CCC* Concordance correlation coefficient, *CT* Computed tomography, *ECV* Extracellular volume, *MRI* Magnetic resonance imaging, *QIR* Quantum iterative reconstruction, *VMI* Virtual monoenergetic image

For Step 2, QIR 4, the QIR level showing the highest agreement and lowest mean bias in Step 1, and VMI at 65 keV were kept constant, while slice thickness/increment were adjusted. The combination of reconstruction parameters with slice thickness 0.4 mm and increment 0.3 mm demonstrated a significant difference compared to the other slice thickness combinations, as well as the highest CCC (0.94, 95% CI 0.87–0.97) and lowest mean bias (0.8, 95% CI 0.0–1.5, LoA -2.5–4.1). For thicker slices, we observed a reduced concordance and a higher mean bias compared to the MRI-based ECV. Table 2 shows a detailed pairwise comparison among the different slice thicknesses*.* Figure 2 shows the concordance scatterplot diagram and the Bland–Altman plot for the 0.4 mm slice thickness reconstruction.

**Table 2:** Step 2: computed tomography-based myocardial extracellular volume (ECV) quantification using quantum iterative reconstruction level 4 and different slice thicknesses

		Pairwise *p*-values for each slice thickness			
Slice thickness (mm)	ECV (%)	0.4	1.5	3.0	5.0
0.4	32.2 ± 4.9				
1.5	33.1 ± 4.9	**< 0.001**			
3.0	32.8 ± 4.8	**< 0.001**	0.109		
5.0	32.8 ± 4.7	0.169	0.361	0.957	
8.0	34.2 ± 4.6	**< 0.001**	0.041	**0.002**	0.011

Paired samples *t*-test. In boldface, significant *p*-values after Bonferroni correction for multiple testing (in this case, 0.05/10 = significance for *p*-values ≤ 0.005)

### Step 3: VMI optimization

The last reconstruction parameter tested was the VMI level of the LIE scans: different VMIs were combined with a slice thickness of 0.4 mm and a QIR level of 4 based on conclusions from Steps 1 and 2. The *t*-test for paired samples showed a significant difference among the lower keV levels, particularly in the range of 45−60 keV, compared to 70 and 90 kV, as detailed in Table 3. Likewise, we noticed a reduction in the mean bias for the VMIs ranging from 45 to 60 keV, with the lowest bias achieved at 45 keV (mean bias 0.6, 95% CI -0.1–1.4, LoA -2.9–4.2). For 40 keV and any keV higher than 60, the mean bias was higher, indicating an overestimation of CT-based ECV over MRI-based ECV. The CCC showed excellent agreement for all the tested VMIs, ranging from 0.93 for 45 keV to 0.94 for 40 keV.

**Table 3:** Step 3: computed tomography-based myocardial extracellular volume (ECV) quantification using quantum iterative reconstruction level 4 and 0.4-mm slice thickness combined with different virtual monoenergetic images (VMIs)

		Pairwise *p*-values for each slice thickness						
VMI	ECV (%)	45 keV	50 keV	55 keV	60 keV	65 keV	70 keV	90 keV
40 keV	32.3 ± 4.9	0.316	0.697	0.756	0.889	0.736	0.735	0.735
45 keV	32.1 ± 4.9		0.114	0.059	0.289	0.544	0.0070	**0.0002**
50 keV	32.2 ± 4.8			0.486	0.007	0.944	**< 0.0001**	**< 0.0001**
55 keV	32.2 ± 4.8				0.080	0.999	**0.0003**	**0.0001**
60 keV	32.2 ± 4.8					0.889	**0.0002**	**0.0001**
65 keV	32.2 ± 5.0						0.585	0.727
70 keV	32.2 ± 4.8							**0.0005**

Paired samples *t*-test. Significant *p*-value after Bonferroni correction for multiple testing (in this case, 0.05/28 = significance for *p*-values ≤ 0.0018) are reported in boldface

**Fig. 3:**
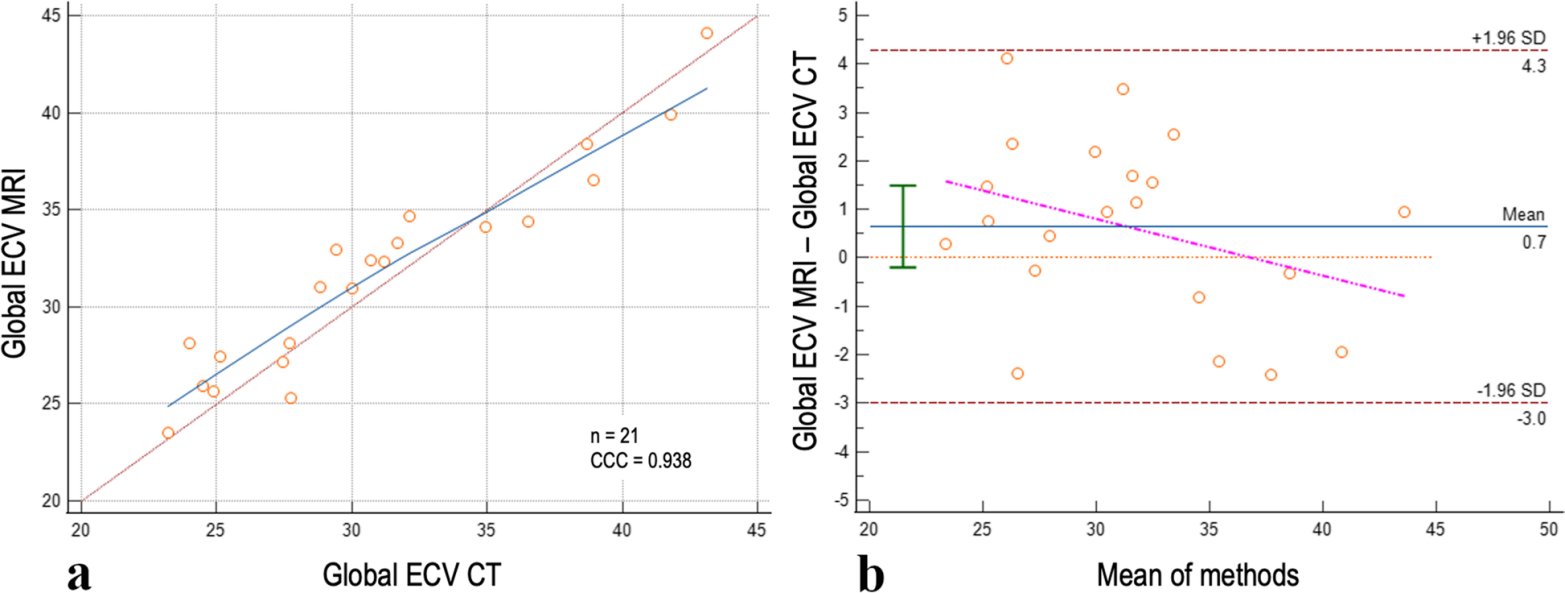
Correlation of ECV between CT and MRI with the best-found combination. Scatterplot (**a**) and Bland–Altman plot (**b**) showing the global ECV correlation between the most optimal combination of CT reconstruction parameters and MRI. The CT parameters were QIR 4, slice thickness 0.4 mm, increment 0.3 mm, and VMI 45 keV. Horizontal lines are drawn at the mean difference (blue), and the limits of agreement (red); in the graph are also displayed the line of equality (orange horizontal line), the regression line of differences (pink line), and the 95% confidence interval of mean difference (green bar). *CCC* Concordance correlation coefficient, *CT* Computed tomography, *ECV* Extracellular volume, *MRI* Magnetic resonance imaging, *QIR* Quantum iterative reconstruction, *VMI* Virtual monoenergetic image

**Fig. 4:**
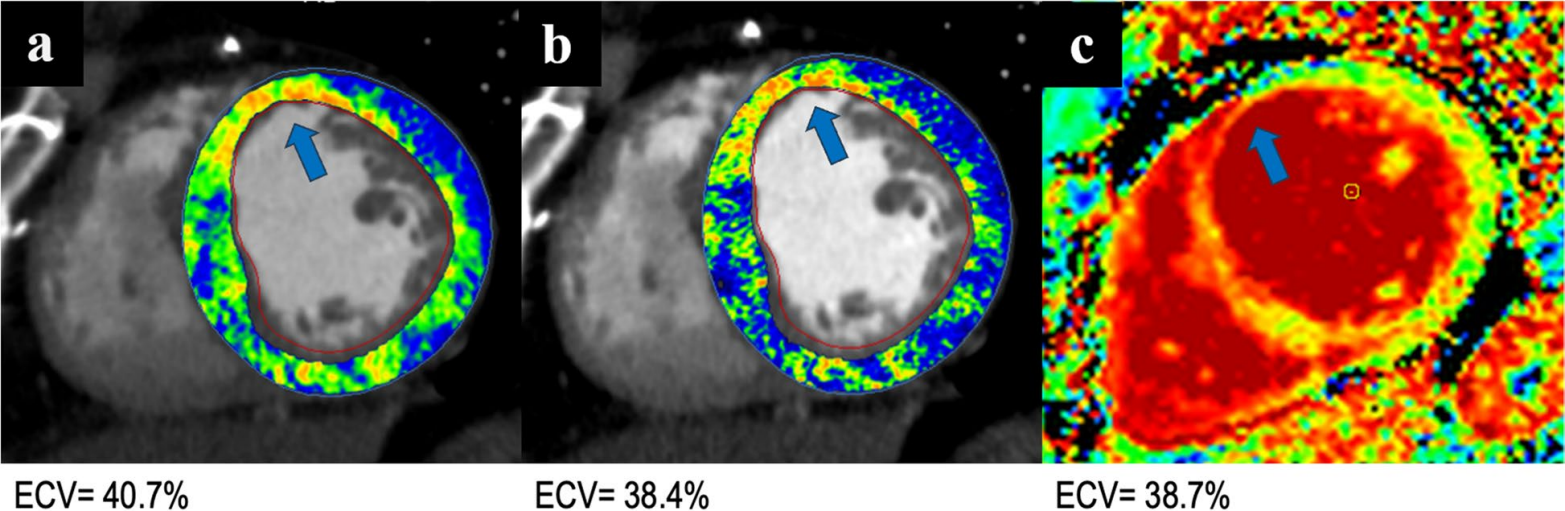
Case of a patient with a history of ischemic cardiomyopathy. CT-based ECV quantification shows anteroseptal 25−50% thickness subendocardial scar and near-transmural apical scar. The CT-based ECV quantification based on the current literature parameters (**a**, QIR 3, slice thickness 1.5 mm, and VMI 65 keV) overestimates the global ECV compared to the quantification performed with the most optimal combination of parameters found in our study (**b,** QIR 4, slice thickness 0.4 mm, and VMI 45 keV), with global ECV values of 40.7% and 38.4%, respectively, *versus* MRI-based ECV of 38.7% (**c**, midventricular MRI-based ECV map)

Hence, according to the stepwise analysis, the best combination of reconstruction parameters was reached when using QIR 4, slice thickness of 0.4 mm with 0.3 mm increment, and 45-keV VMI level. Figure 3 shows the concordance scatterplot and the Bland–Altman plot for the most optimal combination. This combination, compared to the baseline ECV quantification, showed a reduction in the mean bias by 63% and a 6% increase in the agreement with MRI. Figure 4 demonstrates a case example comparing CT-based ECV quantified with the baseline reconstruction parameters, with the most optimal combination based on our results, and MRI-based ECV.

## Discussion

This post hoc analysis of a prospective study investigated the impact of reconstruction parameters on the accuracy of ECV quantification using PCD-CT and the concordance of CT-based ECV with the reference standard MRI-based ECV. The study revealed three major findings.

First, we demonstrated a good-to-excellent correlation between CT- and MRI-based ECV at every investigated QIR strength level, slice thickness, and keV level, highlighting the robustness of CT-based ECV quantification. Second, we demonstrated that adjusting these parameters improves the quantification accuracy of CT-based ECV compared to MRI. In particular, we were able to reduce the mean bias by 63% and increase the concordance with MRI by 6%, when comparing the optimal combination of reconstruction parameters to the baseline proposed in the literature [14, 15]. Third, our systematic methodology enabled the identification of slice thickness as one of the primary parameters significantly influencing ECV quantification and reliability. We chose this systematic step-by-step approach to observe the specific effects of each reconstruction parameter independently and understand the individual contribution to the overall outcome through incremental learning.

The first parameter we evaluated was the strength level of QIR, the iterative reconstruction algorithm specifically introduced for PCD-CT as the commercially available iterative reconstruction algorithms used for EID-CT are not optimal for PCD-CT imaging [19]. In the analyzed scans, QIR strength did not significantly impact the CT-based ECV quantification. Nonetheless, we could notice a slight trend towards increased concordance and decreased mean bias compared to MRI when using higher QIRs. This observation indicates that, unlike conventional iterative reconstruction algorithms, QIR does not affect low-contrast spatial resolution performance, as typical in LIE scan, and does not influence ECV quantification [20]. While conventional iterative reconstruction techniques affect the spatial resolution of low-contrast scans [21], high QIR levels even improve soft-tissue image quality by reducing noise and enhancing contrast-to-noise ratio (CNR) without compromising image texture or CT attenuation values [20].

The second step of our study demonstrated superior accuracy and concordance of CT-based ECV when a thinner slice, 0.4 mm with 0.3 mm increment, was used. This result can be explained by the significant reduction in partial volume averaging, which can lead to significant errors in ECV quantification. When reducing the slice thickness with EID-CT, it should be considered that image noise significantly increases since it is inversely proportional to the square root of the slice thickness [22, 23]. As detailed above, EID-CTs use iterative reconstruction algorithms based on non-linear reconstruction methods, causing a loss in spatial and contrast resolution [20, 21]. Because of its technical limitation, iterative reconstruction cannot compensate for the increased image noise at thinner slices; therefore, to evaluate ECV with EID-CT, thicker slices are usually acquired or reformatted, even though this implies the presence of partial volume averaging. PCD-CT offers the unique possibility to overcome the limitation of partial volume averaging without reducing image quality due to its intrinsic increased spatial resolution and reduced image noise compared to EID-CT [24, 25]. The highest concordance that thinner slices have with MRI quantification of ECV is bound to the higher contrast resolution of myocardial tissue that is achieved with reduced slice thickness and hence reduced *z*-axial partial volume effect [26]. The increased spatial resolution and consequent increase in contrast resolution are fundamental in CT compared to MRI, where the presence of partial volume effect (MOLLI sequences conventionally use 8-mm slice thickness [27]) is balanced by the strength of pixel-wise mapping and the ability of MRI to detect small abnormalities and discriminate low-contrast structures [28]. Even with MRI’s intrinsic high contrast resolution, it is important to note that the use of relatively thick slices may blur the boundary between the myocardium and the blood pool, especially if the myocardial walls are thinned or cardiac motion artifacts are present. In our cohort, such issues prevented the calculation of global MRI-based ECV in six patients.

Finally, we observed a better ECV quantification, with reduced overestimation compared to the MRI reference, at the lower keV range (45 to 60 keV), with 45 keV being the most accurate VMI level. The VMI reconstruction of the LIE scan is pivotal to achieve reliable quantification of ECV, as it delivers both sufficient signal-to-noise ratio and CNR to allow the best alignment with CCTA and consequent extrapolation of quantitative data from the LIE scan-based iodine map. Our results confirm prior evidence demonstrating that lower VMI energies lead to higher diagnostic accuracy compared to higher keV [29, 30]. This difference can be explained by the increased CNR at lower keV and the relative stability of the signal-to-noise ratio throughout different keV levels [30]. As Gutjahr et al. demonstrated this is related to the capability of PCD systems to count all x-ray quanta whose energy is above the lowest threshold so that lower-energy x-rays, bearing most of the low-contrast information, contribute as much to the detector signal as higher energy x-rays [31]. Even though the iodine CNR is highest at 40 keV, studies demonstrated a tendency to prefer slightly higher keV reconstructions for diagnosis to avoid the high image noise present at 40 keV [13]. Euler et al., for example, showed that VMIs in the range 45 to 50 keV represent the best trade-off between objective and subjective image quality [32]; also Albrecht et al. demonstrated that the use of 50-keV VMI provides a reasonable combination of iodine attenuation with preserved image sharpness [33]. This evidence can be explanatory of our results, which show a higher mean bias in Bland–Altman plots for 40 keV compared to the 45–60-keV range, indicating a reduced accuracy in ECV quantification. The role of VMI reconstructions and QIR strength level with PCD-CT has been investigated in other settings and demonstrated a significant impact on CT diagnostic performances in both phantom and clinical studies [34]. This evidence, along with our results, emphasizes the importance to investigate the best acquisition and reconstruction parameters further to achieve the highest diagnostic performance in different clinical settings.

Our study has the following limitations: first, the single-center study design with a relatively small sample size, with a consequent small spectrum of ECV-altering cardiomyopathies investigated; second, the lack of a histological reference; third, the use of MRI-derived synthetic Hct; fourth, the choice of a step-by-step approach rather than a grid approach testing all the possible combinations, which could have introduced a bias; fifth, the use of only a definite range of slice thicknesses and increment; sixth, we did not investigate the impact of different kernels on the accuracy of ECV quantification. Finally, we used a 5-min delayed scan for ECV quantification; however, there are no current studies that investigated the optimal timing for the delayed scan for both ECV quantification and focal scar detection with the novel PCD-CT, an issue that should be investigated in further studies. However, given the novelty of PCD-CT technology, even a limited sample size with the mentioned limitations holds value, particularly considering the unique cohort of patients who underwent same-day MRI and CT imaging.

In conclusion, we demonstrated that an appropriate selection of image reconstruction parameters improves the agreement between ECV values quantified by PCD-CT and MRI. These results highlight the importance of tailoring reconstruction parameters to optimize PCD-CT performance for myocardial tissue characterization.

## Data Availability

The data that support the findings of this study are available from the corresponding authors upon reasonable request.

## Abbreviations

CCC: Concordance correlation coefficient
CCTA: Coronary computed tomography angiography
CI: Confidence interval
CNR: Contrast-to-noise ratio
CT: Computed tomography
ECV: Extracellular volume
EID: Energy-integrating detector
Hct: Hematocrit
LIE: Late iodine enhancement
LoA: Limits of agreement
LV: Left ventricle
MOLLI: Modified Look-Locker inversion-recovery
PCD: Photon-counting detector
QIR: Quantum iterative reconstruction
VMI: Virtual monoenergetic imaging
